# Prospective measurement of the width of cerebrospinal fluid spaces by cranial ultrasound in neurologically healthy children aged 0–19 months

**DOI:** 10.1186/s12887-024-04797-w

**Published:** 2024-05-07

**Authors:** Jozef Fandak, Stefan Markart, Erik P. Willems, Simon Wildermuth, Thomas Frauenfelder, Tim Fischer, Tobias J. Dietrich, Stephan L. Waelti

**Affiliations:** 1 Department of Radiology and Nuclear Medicine, Children’s Hospital of Eastern Switzerland, St. Gallen, 9006 Switzerland; 2 Department of Radiology and Nuclear Medicine, Cantonal Hospital St. Gallen, St. Gallen, Switzerland; 3 Clinical Trials Unit, Biostatistics, Cantonal Hospital St. Gallen, St. Gallen, Switzerland; 4 Department of Diagnostic and Interventional Radiology, University Hospital Zurich, University of Zurich, Zurich, Switzerland

**Keywords:** Cranial ultrasound, Infants, Children, Subarachnoid space, Ventricular width, Benign enlargement of the subarachnoid space (BESS)

## Abstract

**Background:**

Ultrasound (US) is often the first method used to look for brain or cerebrospinal fluid (CSF) space pathologies. Knowledge of normal CSF width values is essential. Most of the available US normative values were established over 20 years ago, were obtained with older equipment, and cover only part of the age spectrum that can be examined by cranial US. This prospective study aimed to determine the normative values of the widths of the subarachnoid and internal CSF spaces (craniocortical, minimal and maximal interhemispheric, interventricular, and frontal horn) for high-resolution linear US probes in neurologically healthy infants and children aged 0–19 months and assess whether subdural fluid collections can be delineated.

**Methods:**

Two radiologists measured the width of the CSF spaces with a conventional linear probe and an ultralight hockey-stick probe in neurologically healthy children not referred for cranial or spinal US.

**Results:**

This study included 359 neurologically healthy children (n_boys_ = 178, 49.6%; n_girls_ = 181, 50.4%) with a median age of 46.0 days and a range of 1–599 days. We constructed prediction plots, including the 5th, 50th, and 95th percentiles, and an interactive spreadsheet to calculate normative values for individual patients. The measurements of the two probes and the left and right sides did not differ, eliminating the need for separate normative values. No subdural fluid collection was detected.

**Conclusion:**

Normative values for the widths of the subarachnoid space and the internal CSF spaces are useful for evaluating intracranial pathology, especially when determining whether an increase in the subarachnoid space width is abnormal.

**Supplementary Information:**

The online version contains supplementary material available at 10.1186/s12887-024-04797-w.

## Background

Enlarged subarachnoid spaces (SASs) and internal cerebrospinal fluid (CSF) spaces in infants and young children have various causes. They may result from hemorrhage, meningitis, hypoxic–ischemic insult, or cerebral atrophy or be idiopathic (e.g., benign enlargement of the subarachnoid space; BESS) [1–3]. In an otherwise healthy child, an apparently enlarged SAS or internal CSF space can lead to difficulty in clinical assessment and unnecessary imaging [4]. Several imaging modalities have been used to measure CSF space width in infants and children, including magnetic resonance imaging (MRI), computed tomography (CT), and ultrasound (US) [4–13]. Normative values cannot be extrapolated from one modality to another because of differences in the parameters, spatial resolution, and planes used [7]. The anterior fontanel is still open in most infants and young children; therefore, US is often the first method used to look for brain or CSF space pathologies. Knowledge of normal values and variations of CSF width is essential. Most of the available values were established over 20 years ago and obtained with older equipment and various probes from small study populations. Moreover, most cover only a part of the age spectrum that can be examined by cranial US [4, 5, 7–9, 13]. The time of closure of the anterior fontanel, the site typically used for US, is highly variable. Therefore, the time when cranial US is still possible is also variable, ranging from a few months to, in rare cases, 20 months [14].

This prospective study used two high-resolution linear probes of different weights and frequencies to determine the normative widths of the SAS and the internal CSF spaces in neurologically healthy, predominantly Caucasian infants and children aged 0–19 months and compared the values between the two US probes. We also assessed whether subdural fluid collections could be delineated with these high-resolution probes. This was of interest because subdural fluid collections may play a role in pathologic enlargement of the subarachnoid space (BESS) and have been described in several trauma settings (particularly birth-related and non-accidental) [15, 16].

## Methods

### Patients

The study was conducted prospectively at a single children’s hospital in central Europe with a predominantly Caucasian population over 12 months (March 2022 to February 2023). Ethical approval was obtained from the local ethics committee. The study was conducted following the Declaration of Helsinki and ICH GCP guidelines. Written informed consent was obtained from the parents of the participants.

Full-term infants and children aged 0–19 months who were referred for a clinically indicated US examination other than a cranial or spinal US (e.g., the abdomen, soft tissues, or hip) were recruited. The children had been delivered without complications and had not required more than routine postnatal care. Exclusion criteria were prematurity (born before 37 weeks of gestation), oxygen deprivation at birth, neonatal intensive care unit admission, a history of being small for gestational age, and a diagnosed genetic disease. All of these conditions can potentially affect the size of the brain and thus the width of the CSF spaces. Additionally, children who had previously undergone US, MRI, or CT of the brain or spine were not included in the study because such examinations may indicate neurological pathology. Body weight, body length, and head circumference were measured on the day of the US examination. Before the examination, the radiologist palpated the anterior fontanel. If it was completely closed, the patient was excluded from the study. If sufficient image quality could not be obtained because the anterior fontanel was still open but too small or the patient was too restless during the examination, the patient was excluded from the study. Children were included in the study only once, even if they underwent multiple US examinations. The US scan for the study was always performed during the first appointment. Patients with choroid plexus cysts were not excluded (all cysts were very small: < 3 mm).

### Ultrasound acquisition

Each US examination was prospectively performed by one of four pediatric radiologists with 7, 8, 13, and 21 years of cranial sonography experience, respectively, using either a LOGIQ E9 or LOGIQ E10 system (GE Medical Systems, Milwaukee, WI, USA). No sedation was used. The scheduled US examination was always performed first. The patient was then placed supine if they were not already in that position. Heated coupling gel was applied to the surface of the probe. The CSF spaces were scanned through the anterior fontanel at the level of the foramina of Monro, perpendicular to the falx cerebri (coronal plane orientation). Care was taken to apply as little pressure as possible. To verify this, the shape of the superior margin of the superior sagittal sinus was observed, which could be upwardly convex or flat but not concave or more compressed. A 6–15-MHz (LOGIQ E9, probe weight 32 g) or 4–20-MHz (LOGIQ E10, probe weight 35 g) linear probe (probes that are routinely used to assess the meningeal space, superior sagittal sinus, and cortical surface) was first used at a frequency of 15 MHz with the manufacturer’s neonatal head preset. The depth was set so that the foramina of Monro were clearly visible. A linear 8–18-MHz (LOGIQ E9, probe weight 15 g) or 6–24-MHz (LOGIQ E10, probe weight 13 g) hockey-stick probe was then used at a frequency of 18 MHz with the manufacturer’s superficial musculoskeletal preset. If the image depth did not reach the foramina of Monro with the hockey-stick probe, scanning was performed in the anterior half of the anterior fontanel. The radiologist who examined the patient recorded one video sequence for the 6–15-MHz probe and one for the hockey-stick probe in the coronal plane through the anterior fontanel, panning from anterior to posterior.

The larger transducer was always used first. In a few cases, the examination was interrupted before the use of the hockey-stick probe because the child was restless. In several cases, the linear probes could not be used because the child had dense, curly hair.

All image datasets were stored in the hospital’s picture archiving and communication system (Dedalus DeepUnity Diagnost 1.1.0.1, Germany).

### Measurements and subdural fluid collection detection

Each measurement was performed by a pediatric radiologist with 13 years of experience and a radiology registrar with 3.5 years of experience. The readers were blinded to the values of the other probe and the results of the other reader. To perform the measurements, the readers used the video sequences recorded by the examining radiologist. All sonographic widths were measured in the coronal plane at the level of the foramina of Monro. The readers selected the appropriate image from the previously recorded videos. The hyperechogenic pia mater covering the brain surface, which is always visible because of the very high spatial resolution, was ignored for the measurements (i.e., measurements were taken up to the cortical surface of the brain). The following widths were measured:

- *Craniocortical width (CCW)*: The shortest vertical width between the inner table of the calvarium and the surface of the subjacent cortical gyrus at the first cortical bulge within 3 cm of the midline falx. The CCW was obtained bilaterally.
- *Minimal interhemispheric width (IHW)*: The smallest horizontal width between the surfaces of the most superior cerebral gyri.
- *Maximal IHW*: The largest horizontal width between the surfaces of the most superior cerebral sulcations.
- *Interventricular width*: The width between the superolateral border of the two lateral ventricles.
- *Frontal horn width*: The width from the inner ventricular margins perpendicular to the maximum convexity of the caudate nuclei to the inferior surface of the corpus callosum. The frontal horn width was obtained bilaterally.

**Fig. 1:**
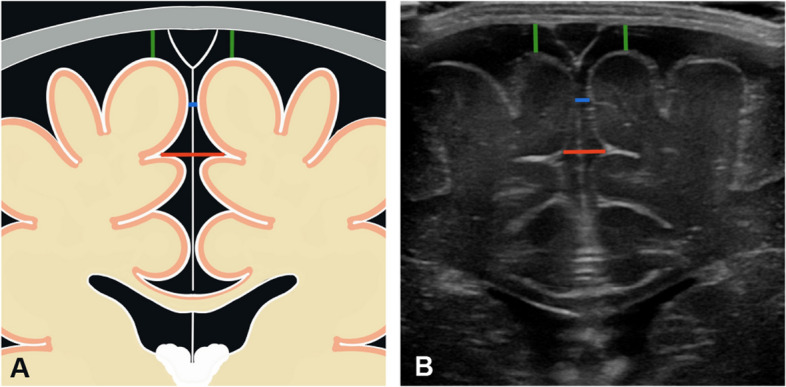
**A** Schematic representation of subarachnoid space measurements. Craniocortical width, right and left (green); minimum interhemispheric width (blue); maximum interhemispheric width (red). **B** Corresponding sonographic image

**Fig. 2:**
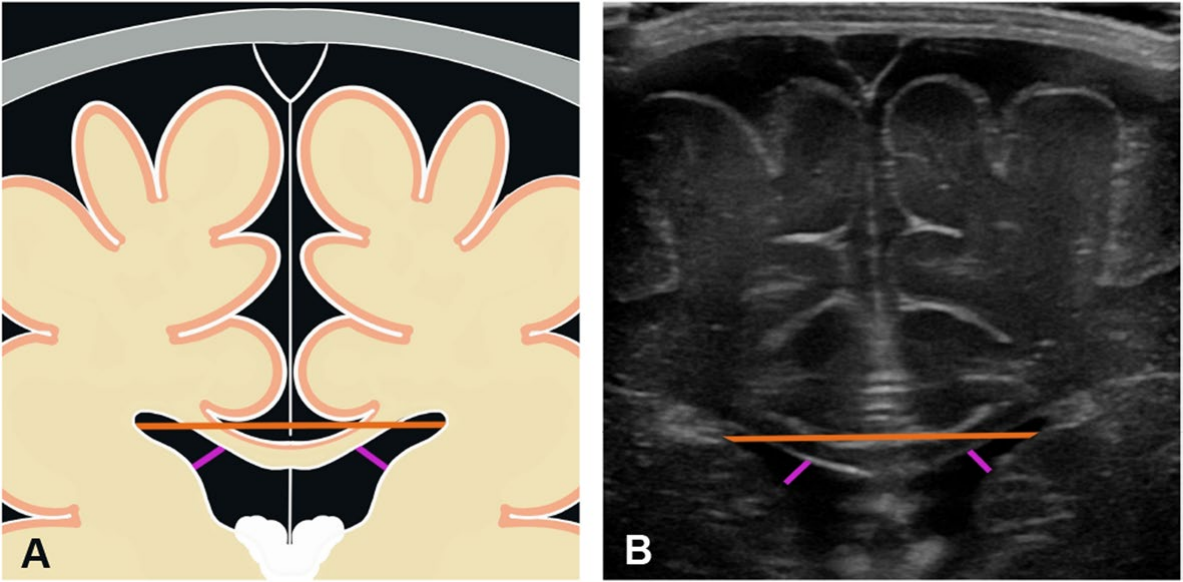
**A** Schematic representation of lateral ventricle measurements. Interventricular width (orange), anterior horn width (purple). **B** Corresponding sonographic image

Figure 1 illustrates the anatomical landmarks and measurements. The measurements are reported in mm to one decimal place. In narrow ventricles, the hyperechogenic ependymal fold was included in the measurement (Fig. 2). In the presence of a cavum septi pellucidi, the frontal horn width was measured up to the leaflet of the septum pellucidum.

Attention was paid to whether a subdural fluid collection was visible. This was coded as present or absent.

### Statistical analysis

#### Reliability of measurements

To quantify the level of agreement between the two readers of the collected US images, intraclass correlation coefficients (ICCs) and Cohen’s κ were calculated for the continuous and discrete variables, respectively. Measurements obtained from the different probes (6–15-MHz probe and hockey-stick probe) and bilaterally (left and right) were evaluated separately, resulting in a total of 13 indices expressing inter-reader reliability.

To assess the level of agreement between the different probes, inter-reader consensus scores were calculated by determining the mean for continuous variables, and the readers always agreed on the one discrete variable (perfect consensus). These inter-reader consensus scores were then used to calculate ICC and Cohen’s κ values, resulting in five inter-probe reliability scores (supplementary material Table S1). Similarly, patient symmetry (the agreement between measurements on the left and right sides) was assessed with inter-reader consensus scores and involved the calculation of three ICCs (supplementary material Table S1).

#### Inferential statistics

To investigate whether sonographic widths were associated with age and sex (as well as the interaction between these two explanatory variables), aggregated consensus scores were calculated. The previous reliability analyses showed that the raw scores could be reasonably aggregated across readers, probes, and sides (given the high ICC values). Therefore, these scores were calculated to remove any remaining pseudoreplication from the data used in our formal analyses. Next, three separate quantile regression models (for the 5th, 50th, and 95th data percentiles) were fitted to these aggregated consensus scores for each variable [17].

To account for possible nonlinear associations with age (human ontogeny is rarely linear over an extended time), we constructed models that included both a linear and a logarithmic term for this explanatory variable. A quadratic association (for example, see Lam et al. [13]) was also considered but found to behave too erratically at the tail ends of the distribution and percentile range, yielding predicted values that were too unrealistically high or low (including negative values) to be biologically meaningful or clinically useful. From the resulting pool of candidate models, those with the most support from the data were selected according to the Akaike information criterion (AIC) [18]. These parsimony-adjusted best-fit models are reported under the Results.

## Results

### Description of dataset

We recruited 359 neurologically healthy children (n_boys_ = 178, 49.6%; n_girls_ = 181, 50.4%). The median age was 46.0 days, with a range of 1–599 days and an interquartile range (IQR) of 35.0–155.5 days. The median body length was 56.0 cm, with a range of 44.0–98.0 cm and an IQR of 52.5–64.0 cm. The median weight was 4.8 kg, with a range of 2.1–13.0 kg and an IQR of 4.0–6.8 kg. The median head circumference was 38.0 cm, with a range of 33.0–49.0 cm and an IQR of 36.5–42.5 cm.

### Inter-reader reliability

Overall, the two readers had a very high level of agreement for all variables of interest (Table 1). The IRR statistics were calculated from the raw data (separately for each side and probe). Interestingly, subdural fluid collection was always scored as “absent,” reflecting perfect inter-reader agreement on this variable; however, calculating a Cohen's κ value was thus not possible due to null variance. Since subdural fluid was never observed in our sample, we excluded it from further analysis.

**Table 1:** Inter-reader reliability (IRR) scores for the variables of interest. Scores were calculated separately for the two probes and the two sides of bilateral variables, resulting in a total of 13 IRR values with associated 95% confidence intervals (CIs)

**Variable**	**ICC**	**Lower 95% CI**	**Upper 95% CI**	**Label**
**Craniocortical width**				
Right: 6–15-MHz probe	0.927	0.911	0.940	Excellent
Right: Hockey-stick probe	0.920	0.903	0.935	Excellent
Left: 6–15-MHz probe	0.901	0.879	0.918	Excellent
Left: Hockey-stick probe	0.907	0.887	0.924	Excellent
**Interhemispheric width (min.)**				
6–15-MHz probe	0.912	0.893	0.928	Excellent
Hockey-stick probe	0.906	0.886	0.923	Excellent
**Interhemispheric width (max.)**				
6–15-MHz probe	0.886	0.862	0.907	Good
Hockey-stick probe	0.907	0.886	0.923	Excellent
**Interventricular width**				
6–15-MHz probe	0.677	0.616	0.729	Moderate
**Frontal horn width**				
Right: 6–15-MHz probe	0.873	0.845	0.895	Good
Left: 6–15-MHz probe	0.858	0.828	0.883	Good
**Variable**	**Cohen’s Κ**	**Lower 95% CI**	**Upper 95% CI**	**Label**
**Subdural hematoma**				
6–15-MHz probe	NA	NA	NA	Perfect
Hockey-stick probe	NA	NA	NA	Perfect

### Inter-probe reliability and patient symmetry

The agreement statistics for the consensus scores of the two probes were consistently excellent. We did not observe any noteworthy systematic difference (Table 1), and reporting separate normative values was thus unnecessary.

Patient sides were symmetrical, and we concluded that separate normative values were not necessary (Table 2).

**Table 2:** Agreement statistics between measurements made on the left and right sides of each patient, quantifying the extent of lateral symmetry in the various structures

Variable	ICC	Lower 95% CI	Upper 95% CI	Label
Craniocortical width: 6–15-MHz probe	0.690	0.632	0.740	Moderate
Craniocortical width: Hockey-stick probe	0.704	0.647	0.752	Moderate
Frontal horn width: 6–15-MHz probe	0.865	0.836	0.889	Good

### Demographic data

The patient-specific variables (age, length, weight, and head circumference) were all highly correlated (Table 3). Therefore, in our subsequent analyses, we considered only patient age because this is the most readily available metric to most radiologists and treating physicians, as well as arguably the most easily and accurately measured metric. Thus, the aggregated consensus scores for the variables of interest are expressed as a function of patient age and gender only.

**Table 3:** Correlation matrix of the continuous candidate explanatory variables. The high correlation coefficients indicate that only one of these variables can be included at a time in any statistical model for the size of the structures of interest

	Age	Length	Weight	Head circumference
Age	1.000			
Length	0.899	1.000		
Weight	0.913	0.932	1.000	
Head circumference	0.902	0.906	0.939	1.000

### Quantile regression models

Model selection based on the AIC suggested that all five outcome variables (CCW, minimal IHW, maximal IHW, interventricular width, and frontal horn width) were best expressed as an additive function of the natural logarithm of age and sex.

**Fig. 3:**
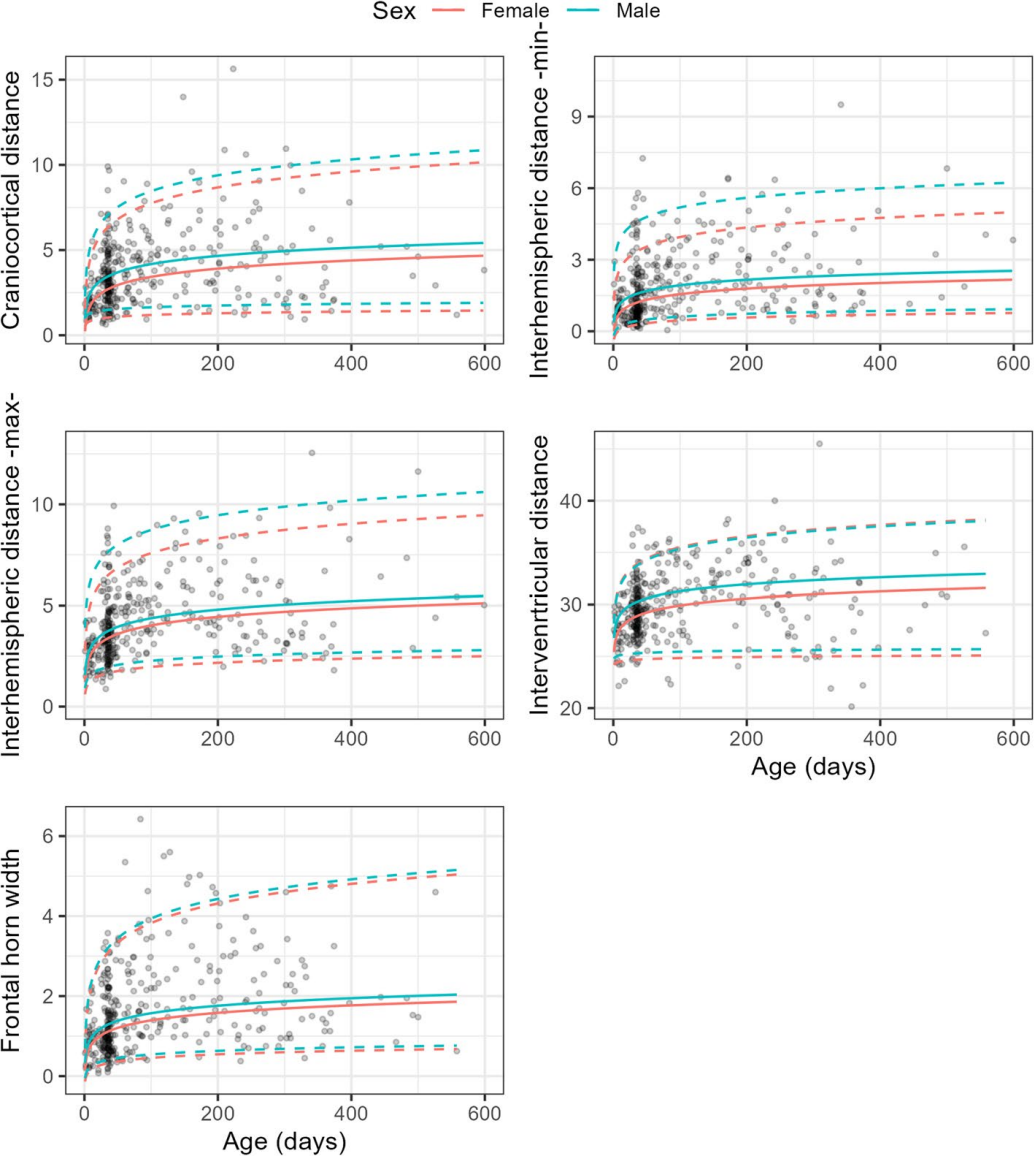
Scatter plots of aggregated data. Each dot represents the aggregated consensus score for one participant. Lines represent predictions from quantile regression models in which the outcome (y-axis, always measured in mm) was expressed as a function of patient age (x-axis) and sex (female = pink, male = blue). Solid lines represent predicted values for the 50th percentile and dashed lines the 5th and 95th percentiles. In the Supplementary Material, we provide an interactive spreadsheet that readers can use to calculate normative values

Prediction plots of the three quantile regressions per variable are shown in Fig. 3.

Additionally, the Supplementary Material provides an interactive spreadsheet that physicians can download and use to calculate normative values for their patients, depending on their age and sex.

We chose not to present the normative values according to age group. This is partly because grouping continuous variables into discrete bins introduces artifacts and always involves an element of arbitrariness. It is also because the age distribution across our patient cohort was very uneven (more patients were in the first few months of life).

## Discussion

We used up-to-date equipment to define contemporary normative values for the widths of the SAS and internal CSF spaces according to age in neurologically healthy, predominantly Caucasian infants and children aged 0–19 months. US is a readily available, inexpensive method without ionizing radiation that does not require sedation or anesthesia. It is ideal for evaluating CSF spaces in infants and children before the closure of the anterior fontanel. Most available studies are more than 20 years old and were performed using older equipment with low spatial resolution and transducers with a frequency of 5–10 MHz [4–9, 13, 19–21]. The significantly improved spatial resolution of today’s US machines warrants the establishment of new, contemporary normative values.

We used linear transducers because they provide higher spatial resolution than convex transducers. We routinely use these along with the standard convex cranial transducer when evaluating SAS pathology, the meninges, the dural sinuses, and the cerebral cortex. The measurements did not differ between the two linear probes so the same normative values can be used for both. Therefore, the weight of the probe seems unimportant; rather, applying minimal pressure is essential.

The size of the anterior fontanel and its resulting usability as a window for US is highly variable. The size an closure timing are influenced by ethnicity (Black infants have larger fontanels than infants of other ethnicities), sex (earlier closure is observed in boys than girls), and other factors [22]. The median age of closure is 13.8 months. By 3 months of age, the anterior fontanel is closed in 1% of infants; by 12 months, it is closed in 38%; and by 24 months, it is closed in 96% [14]. Because the anterior fontanel becomes too small for US before the complete closure of the fontanel, we decided on an upper age limit of 19 months for this study.

The left and right sides appeared symmetrical, and separate normative values did not appear necessary (Table 2). A series of paired t-tests indicated that the width of the frontal horn was significantly greater on the left side than on the right (t = 2.31, df = 354, *p* < 0.05). However, after correcting for multiple comparisons (using the Benjamini–Hochberg procedure), this finding was no longer significant (*p* = 0.065). Moreover, given that the mean difference between the left and right frontal horn widths was 0.07 mm (95% CI = 0.01–0.14 mm; below the measurement accuracy), this can be safely assumed not clinically relevant.

Only one other study, published by Lam et al. [13] in 2001, included children up to 18 months of age. The authors included 278 infants and children and performed the examinations with a 7.5-MHz linear transducer. They found an increase in the SAS width up to 28 weeks of age (approximately 7 months), after which a decrease was observed. Our data do not support this. Unfortunately, the original data from Lam et al. [13]. were no longer available so a detailed statistical comparison was not possible. Libicher et al. [7] examined 89 infants ranging in age from 1 day to 12 months. The correlations between the SAS space width and the infants’ age, weight, and height were poor.

Neither Lam et al. [13] nor Narli et al. [4] found significant differences between sexes. In our study, the values were consistently slightly greater in boys than girls. The results of the study by Lam et al. [13] suggested that ethnicity may influence the SAS width. They found that the SAS was larger in their Chinese population than in Caucasian patients. The patient population at our hospital is approximately 95% Caucasian, 2% Asian, 1% African, and 2% other.

Establishing normative values of the external CSF spaces can facilitate the assessment of infants and children with an increased head circumference (e.g., when an external hydrocephalus or BESS is present) and those with a normal head circumference (e.g., determining whether brain atrophy is present). Additionally, knowing the normal values of the internal CSF spaces can facilitate the identification of internal hydrocephalus.

The normative data presented here may aid in the diagnosis of BESS, which is one cause of macrocephaly in infants with open cranial sutures. BESS can be defined as an increased head circumference (more than two standard deviations above the mean compared with international standards [23] or above the 95th percentile) with a widened SAS and mild or no ventricular dilatation, no other cause for the macrocephaly, and no clinical signs of elevated intracranial pressure [13]. It is typically seen between 3 and 12 months of age, with a mean age of 3.4 months when the head circumference becomes abnormal [24]. It most commonly affects boys, with an incidence of 0.4 per 1,000 live births [25]. Children with BESS are born normocephalic or macrocephalic, and most are developmentally normal. They may have a family history of macrocephaly. BESS is self-limiting; it usually resolves by 18–24 months of age and does not require therapy [26]. The cause of BESS is presumably a combination of infant CSF reabsorption and the concomitant presence of birth-related subdural hemorrhage. CSF is continuously produced and must be reabsorbed. In infants, CSF is reabsorbed through the dural capillaries because of the absence of arachnoid granulations. Blood from birth-related hemorrhage can interfere with this resorption, causing dilation of the subarachnoid space. This imbalance between CSF production and resorption persists until the arachnoid granulations are mature enough to take over CSF resorption in the second half of the first year [15, 16]. To date, no established cut-off values exist for diagnosing BESS [27]. To differentiate normal from dilated ventricular spaces or SASs, we suggest using the 95th percentile from our regression model (Fig. 3, spreadsheet in the Supplementary Materials) as the upper limit.

**Fig. 4:**
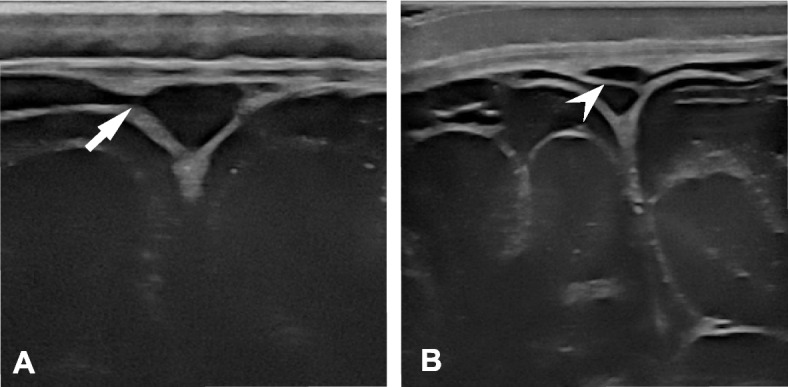
**A** A vein (arrow) that empties into the superior sagittal sinus, resembling a subdural hematoma. **B** A septation (arrowhead) within the superior sagittal sinus, resembling a subdural hematoma

As a secondary question, we wanted to know whether subdural fluid collections (e.g., remnants of birth-related hemorrhage) were visible with the high-resolution US probes. This was not the case, probably because the vast majority of supratentorial birth-associated hemorrhages are localized in the posterior cranium [16] and not at the level of the anterior fontanel where we performed the US. Notably, in our experience, when a radiologist has only single images for reporting (no video sequences), subarachnoid vessels (e.g., bridging veins) or septations in the superior sagittal sinus may mimic a subdural collection (Fig. 4). Because our study used video recording, such pseudopathologies were not a concern. Subdural collections in children younger than 2 years without a medical cause or history of trauma should always raise concern for abusive head injury and be fully evaluated. A controversial association has been identified between an increased SAS depth (e.g., in BESS) and spontaneous or minor trauma-induced subdural hemorrhage due to overdistension of extra-axial blood vessels following brain dislocation [26, 28–34].

This study has several limitations. First, our normative values apply only to full-term infants and children. Infants born prematurely were not included in the study. Second, the measurements we obtained allowed the evaluation of only a limited portion of the subarachnoid space, and isolated frontal or occipital dilatation could not be observed. Third, only a few patients were in the older age groups (Fig. 3). This is mainly because many renovesical pathologies detected in utero are investigated in the first weeks of life, as are screening examinations to detect developmental dysplasia of the hip. Fourth, the inter-reader reliability for the various measurement parameters was probably overestimated because the two readers used the same video sequences. The examining radiologist generated one video sequence using the 6–15-MHz probe and one with the hockey-stick probe for each patient. Due to logistical constraints, two radiologists could not feasibly conduct sonograms on individual patients. Another minor limitation is the variability of the pressure from the probe, which was somewhat unavoidable. In our opinion, this does not reduce the validity of the results because a certain amount of pressure is necessary in real life. Additionally, the slight difference in pressure from one examiner to another and the variation in the child’s agitation corresponds to real-life situations.

## Conclusion

This study provides contemporary normative values for the width of the SAS and internal CSF spaces for high-resolution linear probes in neurologically healthy infants and children aged 0–19 months. We determined that the type of probe used (a conventional linear probe or an ultralight hockey-stick probe) was irrelevant, and the normative values did not differ for the left and right sides. These normative values may be useful in assessing intracranial pathology, particularly in determining whether an increase in the width of the CSF spaces is pathological. Subdural fluid collections were not an incidental finding.

### Supplementary Information

Supplementary Material 1.

Supplementary Material 2.

## Data Availability

The datasets used and/or analyzed during the current study are available from the corresponding author on reasonable request.

## Abbreviations

BESS: Benign enlargement of the subarachnoid space
CCW: Craniocortical width
CSF: Cerebrospinal fluid
CT: Computed tomography
IHW: Interhemispheric width
IQR: Interquartile range
MHz: Megahertz
MRI: Magnetic resonance imaging
SAS: Subarachnoid space
US: Ultrasound
