# Supplementary material for: Prospective measurement of the width of cerebrospinal fluid spaces by cranial ultrasound in neurologically healthy children aged 0–19 months
Source: BMC Pediatr. 2024 May 7;24:315. doi: 10.1186/s12887-024-04797-w (PMC11075241; doi:10.1186/s12887-024-04797-w)
Supplement: Supplementary file 2 — Supplementary Material 2. [file 12887_2024_4797_MOESM2_ESM.docx]

**Inter-probe reliability and patient symmetry**

In the main text of our MS, on the basis of ICC-values we report that no systematic differences were found between the two probes, nor the left and right side within each patient (Table S1). Here we summarize findings from paired t-tests and a Fisher exact test to more formally demonstrate this.

**Table S1** Mean differences between measurements obtained by the 6-15 MHz probe and Hockey-stick probe, or between the left and right side within each patient, respectively. Negative values imply that measurements made by the Hockey-stick probe / on the left side of the patient were larger than those made by the 6-15 MHZ probe /on their right side, and vice versa. For continuous variables, paired t-tests were employed, whereas for the presence/absence of subdural hematomas an odds ratio was estimated using Fisher’s exact test (p-values were adjusted for multiple comparisons using the Benjamini and Hochberg procedure).

| **Comparison of** | **Variable** | **Mean difference** | **Test-statistic**  **(t / odds ratio)** | **p-value** |
| --- | --- | --- | --- | --- |
| Probes | Craniocortical width - right | -0.015 mm | -0.618 | 0.860 |
|  | Craniocortical width - left | -0.033 mm | -1.341 | 0.482 |
|  | Interhemispherical width | 0.001 mm | 0.049 | 1.000 |
|  | Interhemispherical width - max | -0.010 mm | -0.285 | 1.000 |
|  | Subdural hematoma | 0 | 0.000 | 1.000 |
|  |  |  |  |  |
| Sides | Craniocortical width - 6-15 MHz probe | -0.062 mm | -1.029 | 0.609 |
|  | Craniocortical width - Hockey-stick probe | -0.090 mm | -1.478 | 0.482 |
|  | Frontal horn width – 6-15 MHz probe | -0.074 mm | -2.308 | 0.173 |
